# The temporal trends of ST-elevation myocardial infarction mortality according to infarct size and location: insights from the UK National MINAP registry from 2005 to 2019

**DOI:** 10.1093/ehjopen/oeaf111

**Published:** 2025-08-20

**Authors:** Nicholas Weight, Rodrigo Bagur, Nicholas Chew, Sripal Bangalore, Purvi Parwani, Louise Y Sun, Yu Chen Wang, Muhammad Rashid, Mamas A Mamas

**Affiliations:** Keele Cardiovascular Research Group, Centre for Prognosis Research, Institute for Primary Care and Health Sciences, Keele University, Keele ST5 5BH, UK; London Health Sciences Centre, Division of Cardiology, Department of Medicine, Schulich School of Medicine & Dentistry, Western University, London, Ontario, Canada N6A 5A5; Department of Cardiology, National University Heart Centre, National University Health System, Singapore 119074, Singapore; Yong Loo Lin School of Medicine, National University of Singapore, Singapore 117597, Singapore; New York University Grossman School of Medicine, New York, NY 10016, USA; Division of Cardiology, Department of Medicine, Loma Linda University Health, Loma Linda, CA 92354, USA; Division of Cardiothoracic Anesthesiology, Department of Anesthesiology, Perioperative and Pain Medicine, Stanford University School of Medicine, Stanford, CA 94305, USA; Division of Cardiology, Department of Internal Medicine, Asia University Hospital, Taichung 413505, Taiwan; Department of Medical Laboratory Science and Biotechnology, Asia University, Taichung 41354, Taiwan; Keele Cardiovascular Research Group, Centre for Prognosis Research, Institute for Primary Care and Health Sciences, Keele University, Keele ST5 5BH, UK; Department of Cardiovascular Sciences, University of Leicester, Leicester LE1 7RH, UK; National Institute for Health Research (NIHR) Leicester Cardiovascular Biomedical Research Unit, Glenfield Hospital, Leicester LE3 9QP, UK; Keele Cardiovascular Research Group, Centre for Prognosis Research, Institute for Primary Care and Health Sciences, Keele University, Keele ST5 5BH, UK; National Institute for Health and Care Research (NIHR) Birmingham Biomedical Research Centre, Birmingham B15 2TH, UK

**Keywords:** ST-elevation myocardial infarction, Anterior infarction, Non-anterior infarction, Thirty-day mortality, One-year mortality, Troponin assay

## Abstract

**Aims:**

Myocardial infarction size is associated with mortality in ST-elevation myocardial infarction (STEMI). With advances in primary percutaneous coronary intervention (PPCI) and medical therapy, whether this relationship has changed over time is unclear.

**Methods and results:**

Patients with STEMI in the UK from 2005 to 2019 were included from the national AMI MINAP registry, with mortality linkage to 2021. Primary outcomes were all-cause mortality at 30 days and 1 year according to infarct size, using Cox regression models. Infarct size was stratified by Tertiles (T1–3) of peak troponin level (T1, smallest; T3, largest), across the early (2005–09), middle (2010–14), and late (2015–19) periods. Subgroup analyses assessed the relationship according to infarct location (anterior vs. non-anterior). A total of 177 214 STEMI patients were included. Adjusted 30-day mortality risk according to infarct size was highest in the early period (aHR: 1.32, 1.21–1.45, *P* < 0.001), compared to middle (1.12, 1.04–1.20, *P* = 0.002) and late study periods (1.05, 0.96–1.14, *P* = 0.299). The relationship between infarct size and 30-day mortality was significant for patients with anterior STEMI in early (1.39, 1.22–1.57, *P* < 0.001) but not middle or late periods, while remained significant for non-anterior infarction until the late period (early, 1.28, 1.13–1.45, *P* < 0.001; middle, 1.17, 1.06–1.29, *P* = 0.002; late, 1.09, 0.96–1.24, *P* = 0.180).

**Conclusion:**

We observed an independent relationship between infarct size and STEMI mortality, strongest between 2005 and 2009, which reduced over time, becoming non-significant in the 2015–19 period. This association diminished more rapidly for patients with anterior STEMIs. These findings underscore the potential role of contemporary revascularization, systems of care, and guideline-directed medical therapy in reducing STEMI-related mortality.

## Graphical Abstract

**Graphical Abstract:**
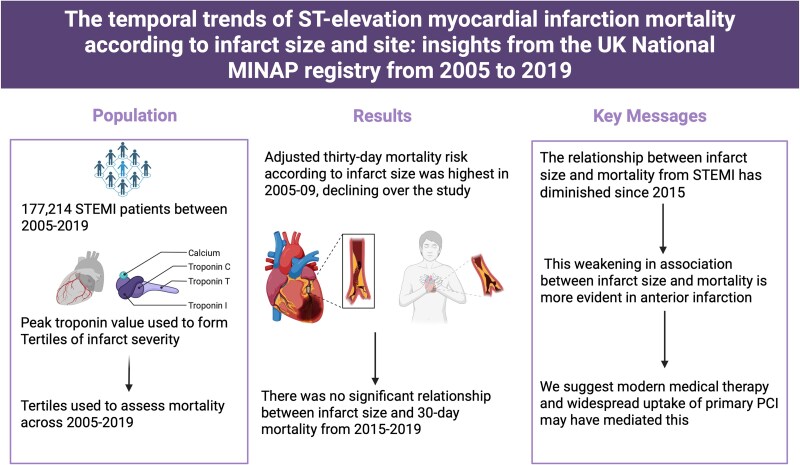

## Introduction

ST-elevation myocardial infarction (STEMI) remains a significant cause of morbidity and premature mortality worldwide.^1^ Contemporary guideline-directed medical therapy (GDMT), systems of care, and widespread primary percutaneous coronary intervention (PPCI) have led to significant improvements in mortality, particularly in the reduction of post-MI complications from STEMI.^2,3^

However, prognosis following STEMI remains variable, depending on factors such as time to definitive reperfusion and culprit vessel, infarct size, and infarct location, with anterior infarcts particularly associated with poorer outcomes.^4^ Myocardial infarct size has been assessed in a range of ways, including peak biomarker level [troponin T or high-sensitivity troponin (hs-TnT or hs-TnT)]^5,6^ as well as cardiac magnetic resonance imaging (CMRI) or technetium-99 m sestamibi single-photon emission computed tomography (SPECT).^7^ Historically, larger infarct size by either cardiac biomarker or imaging has predicted poorer left ventricular function and mortality.^8^ However, with continued advances in modern PPCI networks and techniques, alongside advances in GDMT, the relationship between infarct size according to biomarker rise and mortality, and whether it has changed over time, remains unclear.

We therefore investigated the relationship between STEMI infarct size according to peak troponin value and 30-day and 1-year mortality, and whether this relationship varied according to infarct location, using a national registry with comprehensive all-cause mortality linkage between 2005–19.

## Methods

### Study design

We conducted a retrospective cohort study using prospectively collected Myocardial Ischaemia National Audit Project (MINAP) data linked to the Office for National Statistics (ONS) and Hospital Episode Statistics (HES). MINAP is a prospective national registry of patients with acute coronary syndrome (ACS) admitted to 230 hospitals in the UK. It includes data on patient demographics, clinical characteristics, comorbidities, pharmacotherapy, management, and in-hospital outcomes, on ∼90 000 patients admitted with ACS per year. The ONS is the independent provider of mortality statistics in the UK, collecting data on all deaths registered in the UK using *International Classification of Diseases, Tenth Revision (ICD-10)* codes and cause of death from the Medical Certificate of Cause of Death (MCCD). The ONS allows for longitudinal mortality ascertainment up to July 2021, in conjunction with MINAP’s short-term, in-hospital outcomes.

### Study population

**Figure 1:**
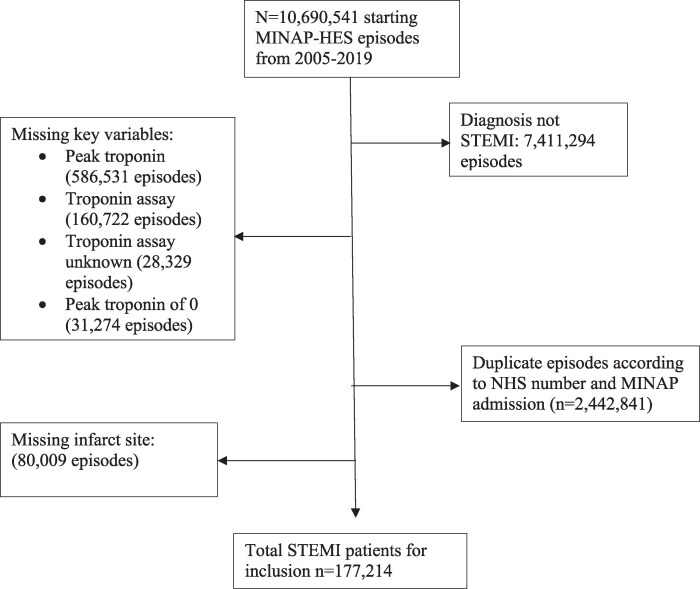
STROBE diagram detailing study exclusion criteria from total starting registry population.

We included all patients admitted to hospitals in the UK between January 2005 and March 2019 with a primary diagnosis of STEMI, who had valid peak troponin values and assay type [troponin I, troponin T, high-sensitivity troponin I (hs-TnI), and high-sensitivity troponin T (hs-TnT)], as well as data regarding infarct location. Patients missing this data, alongside any missing outcome data such as in-hospital mortality, major adverse cardiovascular events, cause of death, missing National Health Service (NHS) number, or inconsistent mortality dates, were excluded from the study (*Figure 1*). Patients were stratified according to infarct size as defined by tertiles of peak troponin values, calculated for each individual assay for each individual study year. Therefore, each tertile contains the corresponding tertile for each individual assay used over that study period, meaning that, for example, the highest overall tertile will comprise the highest tertiles of troponin T and I and hs-TnT and hs-TnI. Tertile sizes are not completely even due to clustering of values at the maximum recorded range for certain assays; for patients at the maximum recorded level for an assay, these were always included in Tertile 3, which was our highest tertile, reflective of the most significant infarct size. To enable assessment of temporal trends over the course of the study, we defined 2005–09 as early, 2010–14 as middle, and 2015–19 as the late study period. To assess the impact of infarct location on mortality, we stratified patients into anterior and non-anterior infarct groups, the latter being a composite of inferior, posterior, lateral, and indeterminate infarcts. Infarct site is determined in the MINAP registry as the territory showing the most extensive ST-segment changes on admission ECG.

STEMI diagnosis was made by clinicians according to the presenting history, clinical examination, and investigations as per European Society of Cardiology (ESC) guidelines. The first admission with STEMI over our study period for each patient is included, with duplicate records or readmissions over the study period identified and removed using the date of admission and NHS number.

### Outcomes

The primary outcomes were all-cause mortality, assessed at 30 days and 1 year according to infarct size, for each of the pre-determined study periods (2005–09, 2010–14, and 2015–19). Secondary outcomes were 30-day and 1-year all-cause mortality according to infarct location, stratified by infarct size. Additionally, we assessed the relationship between peak troponin value as a continuous variable and 30-day and 1-year mortality across the early, middle, and late study periods.

### Statistical analysis

Continuous variables were summarized using mean and standard deviation if normally distributed and median and interquartile ranges (IQR) if their distribution was not normal. Normality of distribution was assessed using Shapiro–Wilk test, data were compared using Student’s *t-*test if normally distributed, and Wilcoxon rank sum test if not normally distributed. Categorical variables were compared using the Pearson *χ*^2^ test and summarized as percentages (%). Multiple imputation with 10 imputed datasets was used to deal with missing data. Multiple imputation with chained equations (MICE) is the best practice when dealing with missing data and can provide unbiased estimates even with high levels of missingness, and some protection when data are missing not at random.^9^

Multivariable Cox regression was performed on ten imputed datasets, using Rubin’s rules^10^ to generate adjusted hazard ratios (aHR) with 95% confidence intervals (95% CI) for mortality at 30 days and 1 year, for tertiles according to peak troponin value as a surrogate for infarct size, for each of the three time periods. Our model was adjusted for the following characteristics: age, sex, year of admission, hospital region, heart rate, blood pressure, comorbid conditions (hypertension, diabetes mellitus, history of asthma, or COPD, history of CVA or PVD, hypercholesterolaemia, family history of coronary artery disease, smoking history, chronic renal failure, previous AMI, angina, previous PCI, and previous CABG), whether taking warfarin at admission, and cardiac arrest. Due to concerns over collinearity with peak troponin value, we elected not to adjust for LV systolic function as a covariate. Hazard ratios shown are from comparison of patients with the smallest infarct size (i.e. lowest troponin tertile). The Kaplan–Meier survival curves and Schoenfeld residuals demonstrated that the proportional hazard assumption was not violated.

Subgroup analyses were undertaken using the ‘margins’ command on the imputed dataset, after applying a logistic regression model with 30-day and 1-year mortality as the outcome variables, adjusted for the previously mentioned covariates, additionally with an interaction term for peak troponin tertile (1, 2, and 3) and temporal group (2005–09, 2010–14, and 2015–19). This was then shown in graph form using the ‘marginsplot’ command. All analyses were undertaken on Stata 18.0. A personal ‘BioRender’ licence was used to create the graphical abstract. Figures were created using a Keele University Institutional ‘GraphPad’ account.

## Results

After applying relevant exclusion criteria (*Figure 1*), a total of 177 214 patients admitted with a primary diagnosis of STEMI were studied, of whom 41 066 were from the 2005–09, 77,343 from 2010–14, and 58 805 from 2015–19 periods. For ease of reference, 2005–09 was referred to as early, 2010–14 as middle, and 2015–19 as late periods, and Tertiles 1–3 will be indicated as T1–3.

### Baseline clinical characteristics according to infarct size and time period

Baseline patient characteristics are summarized in *Table 1*. Throughout the study period, patients with larger infarcts were less likely to be female: early (T1: 33% vs. T3: 26%), middle (T1: 31% vs. T3: 26%), and late (T1: 26% vs. T3: 24%). Similarly, a history of previous myocardial infarction was less common among patients with larger infarcts: early (T1: 16% vs. T3: 11%), middle (T1: 13% vs. T3: 10%), and late (T1: 11% vs. T3: 8%). They more often had anterior infarction: early (T1: 42% vs. T3: 46%), middle (T1: 41% vs. T3: 45%), and late (T1: 40% vs. T3: 44%).

**Table 1:** Demographic comparison of patients admitted with STEMI, stratified by infarct size (according to peak troponin tertile)

Variables	Admission with STEMI 2005–09 (early)			Admission with STEMI 2010–14 (middle)			Admission with STEMI 2015–19 (late)		
	Tertile 1 (smallest peak troponin) (*n* = 13 667)	Tertile 2 (*n* = 13 471)	Tertile 3 (highest peak troponin) (*n* = 13 929)	Tertile 1 (smallest peak troponin) (*n* = 25 782)	Tertile 2 (*n* = 24 833)	Tertile 3 (highest peak troponin) (*n* = 26 728)	Tertile 1 (smallest peak troponin) (*n* = 19 480)	Tertile 2 (*n* = 19 698)	Tertile 3 (highest peak troponin) (*n* = 19 627)
Age, years, median (IQR)	67.3 (56.6–77.8)	66.8 (56.5–77.1)	66.6 (56.7–76.8)	65.9 (55.5–77.1)	65.6 (55.6–76.3)	65.7 (55.7–76.6)	65.5 (55.5–76.1)	66.4 (56.2–76.8)	65.6 (55.8–75.8)
Female (%)	4548/13 667 (33)	4073/13 471 (30)	3578/13 928 (26)	7922/25 782 (31)	7050/24 833 (28)	7032/26 728 (26)	5126/19 480 (26)	5594/19 698 (28)	4664/19 627 (24)
BMI, median (IQR)	26.4 (23.6–29.8)	26.6 (23.8–29.8)	26.4 (23.7–29.6)	26.8 (23.8–30.1)	26.7 (24.0–30.1)	26.7 (24–30.0)	27.0 (24.2–30.4)	27.1 (24.2–30.4)	27.0 (24.2–30.4)
Ethnicity, White (%)	N/A	N/A	N/A	15 104/16 570 (91)	15 847/17 162 (92)	17 310/19 130 (90)	14 257/17 172 (83)	15 731/17 556 (90)	16 408/18 227 (90)
Ethnicity, Asian (%)	N/A	N/A	N/A	977/16 570 (6)	826/17 162 (5)	1036/19 130 (5)	1754/17 172 (10)	1131/17 556 (6)	1053/18 227 (6)
Ethnicity, Black (%)	N/A	N/A	N/A	189/16 570 (1)	112/17 162 (1)	136/19 130 (1)	264/17 172 (2)	113/17 556 (1)	138/18 227 (1)
Ethnicity, Mixed (%)	N/A	N/A	N/A	300/16 570 (2)	377/17 162 (2)	648/19 130 (3)	897/17 172 (5)	581/17 556 (3)	628/18 227 (3)
Basal crepitations (%)	N/A	N/A	N/A	1325/14 626 (9)	1273/14 296 (3)	1477/14 061 (11)	1274/17 850 (7)	1115/18 127 (6)	1346/18 418 (7)
Pulmonary oedema (%)	N/A	N/A	N/A	588/14 626 (4)	487/14 296 (3)	686/14 061 (5)	488/17 850 (3)	439/18 127 (2)	719/18 418 (4)
Cardiogenic shock (%)	N/A	N/A	N/A	474/14 626 (3)	366/14 296 (3)	463/14 061 (3)	733/17 850 (4)	449/18 127 (2)	649/18 418 (4)
Previous smoker (%)	3717/12 723 (29)	3826/12 617 (30)	3758/13 090 (29)	6734/24 545 (27)	6620/23 491 (28)	6995/25 838 (27)	4751/18 447 (26)	4930/18 835 (26)	5047/18 968 (27)
Current smoker (%)	4873/12 723 (38)	4777/12 617 (38)	5176/13 090 (40)	9377/24 545 (38)	9343/23 941 (39)	10 014/25 838 (39)	6922/18 447 (38)	6933/18 835 (37)	6929/18 968 (37)
CCF (%)	340/12 817 (3)	246/12 719 (2)	201/13 149 (2)	513/24 746 (2)	358/23 910 (2)	437/25 548 (2)	334/18 590 (2)	278/18 264 (2)	289/17 359 (2)
Hypercholesterolaemia (%)	3960/12 800 (31)	3815/12 656 (30)	3975/13 028 (31)	7807/24 524 (32)	7103/23 737 (30)	7558/25 335 (30)	5984/18 674 (32)	5424/18 417 (29)	5098/17 438 (29)
Cerebrovascular disease (%)	789/12 777 (6)	661/12 667 (5)	747/13 096 (6)	1455/24 642 (6)	1257/23 828 (5)	1349/25 534 (5)	953/18 607 (5)	803/18 283 (4)	713/17 360 (4)
History of angina (%)	2366/13 065 (18)	1964/12 963 (15)	1801/13 346 (13)	3385/24 681 (14)	2650/23 873 (11)	2826/25 576 (11)	1821/18 579 (10)	1683/18 300 (9)	1415/17 360 (8)
Peripheral vascular disease (%)	433/12 500 (3)	390/12 301 (3)	359/12 531 (3)	801/24 521 (3)	671/23 741 (3)	736/25 458 (3)	543/18 585 (3)	390/18 283 (2)	396/17 358 (2)
Chronic renal failure (%)	360/12 810 (3)	313/12 711 (2)	296/13 126 (2)	709/24 595 (3)	613/23 763 (3)	665/25 442 (3)	531/18 579 (3)	440/18 158 (2)	473/17 293 (3)
Hypertension (%)	5805/13 166 (44)	5465/13 013 (42)	5695/13 419 (42)	11 008/24 952 (44)	10 131/24 068 (42)	10 680/25 672 (42)	8565/18 854 (45)	7922/18 576 (43)	7243/17 529 (41)
Asthma/COPD (%)	1663/12 515 (13)	1456/12 380 (12)	1332/12 525 (11)	3167/24 712 (13)	2741/23 850 (11)	2810/25 532 (11)	2332/18 633 (13)	2302/18 312 (13)	1914/17 379 (11)
Family history of CAD (%)	4180/11 235 (37)	4232/11 310 (37)	4413/11 900 (37)	6987/22 009 (32)	7562/21 459 (35)	8387/23 085 (36)	4660/16 122 (29)	5230/16 185 (32)	5021/15 499 (32)
Previous AMI (%)	2076/13 243 (16)	1672/13 091 (13)	1460/13 459 (11)	3275/25 017 (13)	2440/23 995 (10)	2615/25 632 (10)	2140/18 713 (11)	1725/18 388 (9)	1434/17 407 (8)
Previous PCI (%)	778/12 934 (6)	697/12 917 (5)	701/13 336 (5)	1778/24 759 (7)	1478/23 892 (6)	1492/25 583 (6)	1609/18 697 (9)	1310/18 338 (7)	1083/17 384 (6)
Previous CABG (%)	429/12 959 (3)	336/12 951 (3)	213/13 334 (2)	796/24 790 (3)	583/23 905 (2)	495/25 592 (2)	503/18 684 (3)	416/18 316 (2)	271/17 383 (2)
Heart rate, b.p.m., median (IQR)	78 (65–92)	76 (64–90)	75 (63–90)	76 (65–90)	75 (64–89)	76 (64–90)	77 (65–89)	77 (65–90)	76 (65–89)
Systolic blood pressure, median (IQR)	138 (120–157)	135 (117–155)	135 (116–154)	132 (115–151)	130 (112–149)	129 (110–148)	131 (114–150)	130 (114–150)	129 (111–148
Anterior infarct (%)	5801/13 667 (42)	4993/13 471 (37)	6354/13 928 (46)	10 456/25 782 (41)	9530/24 833 (38)	12 027/26 728 (45)	7869/19 480 (40)	7551/19 698 (38)	8628/19 627 (44)
Non-anterior infarct (%)	7866/13 667 (58)	8478/13 471 (63)	7574/13 928 (54)	15 326/25 782 (59)	15 404/24 833 (62)	14 701/26 728 (55)	11 611/19 480 (60)	12 147/19 696 (62)	10 999/19 627 (56)
Good LV function (%)	2885/9034 (32)	3030/9199 (33)	2366/9579 (25)	8574/21 148 (41)	8340/21 051 (40)	6108/21 044 (29)	7145/17 227 (41)	7503/17 218 (44)	5561/17 692 (31)
Moderate LVSD (%)	1437/9034 (16)	1991/9199 (22)	2902/9579 (30)	4911/21 148 (23)	6094/21 051 (29)	7574/21 044 (36)	5870/17 227 (34)	6292/17 218 (37)	8345/17 692 (47)
Severe LVSD (%)	421/9034 (5)	457/9199 (5)	830/9579 (9)	1359/21 148 (6)	1403/21 051 (7)	2223/21 044 (11)	1338/17 227 (8)	1229/17 218 (7)	2264/17 692 (13)
Cardiac arrest (%)	1100/13 445 (8)	1264/13 290 (10)	1488/13 637 (11)	2525/25 367 (10)	2637/24 662 (11)	2775/26 488 (10)	2475/19 433 (13)	2263/19 511 (12)	2330/19 465 (12)
Troponin assay									
Troponin I	8075/13 667 (59)	7834/13 471 (58)	8305/13 928 (60)	12 341/25 782 (48)	11 462/24 833 (46)	13 219/26 728 (49)	3955/19 480 (20)	4196/19 698 (21)	4078/19 627 (21)
Troponin T	5592/13 667 (41)	5637/13 471 (42)	5623/13 928 (40)	4735/25 782 (18)	4728/24 833 (19)	4739/26 728 (18)	1283/19 480 (7)	1289/19 698 (7)	1288/19 627 (7)
hs-TnI	N/A	N/A	N/A	7888/25 782 (31)	7885/24 833 (32)	7889/26 728 (30)	9955/19 480 (51)	9943/19 698 (50)	9952/19 627 (51)
hs-TnT	N/A	N/A	N/A	818/25 782 (3)	758/24 833 (3)	881/26 728 (3)	4287/19 480 (22)	4270/19 698 (22)	4309/19 627 (22)

CABG, coronary artery bypass graft; LVSD, left ventricular systolic dysfunction; CAD, coronary artery disease; COPD, chronic obstructive pulmonary disease; MI, myocardial infarction; CCF, congestive cardiac failure; BMI, body mass index; GRACE, global registry of acute coronary events; IQR, interquartile range. Admission to the cardiology ward is a composite of admission to the coronary care unit (CCU) and general cardiology ward. Chronic renal failure is recorded in MINAP as those with serum creatinine chronically elevated above 200 µmol/L. Non-anterior infarct is a composite of inferior, posterior, lateral, and indeterminate infarct sites. Early study period refers to 2005–09, middle study period to 2010–14, and late study period to 2015–19.

### Management strategy according to infarct size and time period

Patients with larger infarcts more often received P2Y12 inhibitors throughout all time periods: early (T1: 86% vs. T3: 90%), middle (T1: 94% vs. T3: 95%), and late (T1: 94% vs. T3: 96%). Prescription of aldosterone antagonists increased significantly across the study: early (T1: 3% vs. T3: 7%), middle (T1: 7% vs. T3: 12%), and late (T1: 9% vs. T3: 20%) (*Table 2*). The proportion of patients undergoing primary PCI increased significantly over the study period: early (T1: 43% vs. T3: 52%), middle (T1: 74% vs. T3: 85%), and late (T1: 92% vs. T3: 97%).

**Table 2:** Management strategy and clinical outcome comparison for patients admitted with STEMI, stratified by infarct size (according to peak troponin tertile)

Variables	Admission with STEMI 2005–09 (early)			Admission with STEMI 2010–14 (middle)			Admission with STEMI 2015–19 (late)		
	Tertile 1 (smallest peak troponin) (*n* = 13 667)	Tertile 2 (*n* = 13 471)	Tertile 3 (highest peak troponin) (*n* = 13 929)	Tertile 1 (smallest peak troponin) (*n* = 25 782)	Tertile 2 (*n* = 24 833)	Tertile 3 (highest peak troponin) (*n* = 26 728)	Tertile 1 (smallest peak troponin) (*n* = 19 480)	Tertile 2 (*n* = 19 698)	Tertile 3 (highest peak troponin) (*n* = 19 627)
Warfarin (%)	358/12 229 (3)	404/12 241 (3)	518/12 553 (4)	778/21 841 (4)	741/21 938 (3)	914/23 836 (4)	460/15 670 (3)	496/16 073 (3)	631/15 605 (4)
Glycoprotein 2b/3a inhibitor (%)	1791/12 490 (14)	2069/12 525 (17)	2572/12 862 (20)	4412/22 551 (20)	4954/22 435 (22)	5659/24 153 (23)	2983/16 852 (18)	2243/16 452 (14)	2559/15 902 (16)
IV nitrate (%)	2796/12 256 (23)	2750/12 269 (22)	3332/12 613 (26)	5298/21 852 (24)	5300/21 954 (24)	5464/23 831 (23)	4345/15 657 (28)	4123/16 085 (26)	3939/15 605 (25)
MRA (%)	122/3876 (3)	156/3830 (4)	269/4060 (7)	1440/21 992 (7)	1742/21 923 (8)	2947/23 737 (12)	1463/15 710 (9)	1829/16 194 (11)	3089/15 714 (20)
Aspirin (%)	13 252/13 567 (98)	13 149/13 422 (98)	13 641 (98)	25 280/25 735 (98)	24 473/24 792 (99)	26 349/26 695 (99)	19 192/19 455 (99)	19 444/19 683 (99)	19 355/19 605 (99)
P2Y12 inhibitor (%)	11 406/13 190 (86)	11 563/13 075 (88)	12 045/13 450 (90)	24 009/25 607 (94)	23 625/24 720 (96)	25 103/26 426 (95)	18 350/19 458 (94)	18 961/19 688 (96)	18 897/19 621 (96)
Statins (%)	11 148/13 525 (82)	11 475/13 410 (86)	11 853/13 822 (86)	21 920/25 715 (85)	21 718/24 782 (88)	23 618/26 651 (89)	17 430/19 445 (90)	17 988/19 664 (91)	18 173/19 606 (93)
ACE inhibitors/ARB (%)	10 461/13 504 (77)	10 844/13 378 (81)	11 279/13 794 (82)	20 899/25 700 (81)	20 939/24 772 (85)	22 826/26 646 (86)	16 755/19 446 (86)	17 469/19 657 (89)	17 565/19 596 (90)
Beta-blockers (%)	10 023/13 504 (74)	10 450/13 385(78)	10 982/13 795 (80)	2 207 625 727 (86)	22 324/24 797 (90)	24 224/26 675 (91)	17 428/19 454 (90)	18 170/19 674 (92)	18 408/19 601 (94)
Primary Percutaneous coronary intervention (%)	2840/13 312 (21)	3503/13 161 (26)	4324/13 422 (32)	18 969/25 769 (74)	19 807/24 822 (80)	22 580/26 708 (85)	17 803/19 456 (92)	18 212/19 657 (93)	18 978/19 613 (97)
CABG surgery (%)	202/11 386 (2)	158/11 431 (1)	107/11 985 (1)	321/21 375 (2)	241/21 250 (1)	198/23 737 (1)	100/11 773 (1)	119/12 888 (1)	85/13 180 (1)
Revascularization (CABG surgery/PCI) (%)	6082/13 624 (45)	6360/13 444 (47)	7343/13 899 (53)	19 201/25 769 (75)	19 982/24 822 (81)	26 708/26 708(85)	17 845/19 456 (92)	18 256/19 657 (93)	19 004/19 614 (97)
Thrombolysis (%)	5676/13 312 (43)	5808/13 161 (44)	7026/13 422 (52)	1570/25 656 (6)	1337/24 712 (5)	1753/26 645 (7)	137/19 452 (1)	134/19 640 (1)	119/19 599 (1)
Inpatient mortality (%)	797/13 667 (6)	804/13 471 (6)	1005/13 928 (7)	1220/25 782 (5)	1123/24 833 (5)	1405/26 728 (5)	836/19 480 (4)	658/19 698 (3)	848/19 627 (4)
Thirty-day mortality (%)	958/13 667 (7)	993/13 471 (7)	1200/13 928 (9)	1597/25 782 (6)	1464/24 833 (6)	1816/26 728 (7)	1080/19 480 (6)	889/19 698 (5)	1088/19 627 (6)
One-year mortality **(%)**	1826/13 667 (13)	1694/13 471 (13)	1965/13 928 (14)	2923/25 782 (11)	2512/24 833 (10)	3134/26 728 (12)	1814/19 480 (9)	1565/19 698 (8)	1877/19 627 (10)
Reinfarction (%)	432/13 122 (3)	387/12 970 (3)	369/13 358 (3)	378/24 876 (2)	333/23 852 (1)	344/25 734 (1)	231/18 755 (1)	174/18 316 (1)	189/17 222 (1)
Major bleeding (%)	233/13 385 (2)	201/13 205 (2)	287/13 582 (2)	249/25 463 (1)	243/24 486 (1)	284/26 486 (1)	113/19 219 (1)	123/19 381 (1)	113/19 276 (1)

IV, intravenous; MRA, mineralocorticoid receptor antagonist; ACE, angiotensin-converting enzyme; ARB, angiotensin receptor blockers; CABG, coronary artery bypass graft; PCI, percutaneous coronary intervention; MACE, major adverse cardiovascular events. MACE is defined as a composite endpoint of in-hospital death and reinfarction. *Chronic kidney disease* is recorded in MINAP as those with serum creatinine chronically elevated above 200 µmol/L. Thrombolysis refers to intravenous administration of thrombolytic agents, not catheter-based. Early study period refers to 2005–09, middle study period to 2010–14, and late study period to 2015–19.

### Unadjusted mortality outcomes according to infarct size and time period

**Figure 2:**
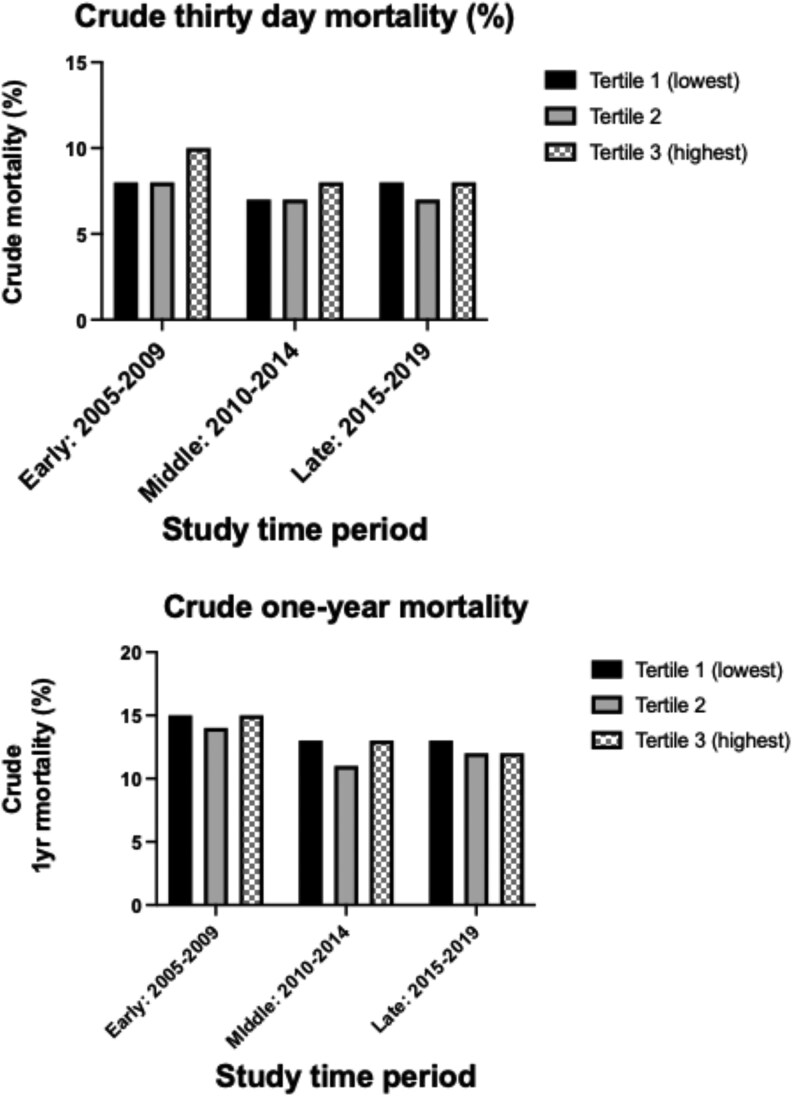
Unadjusted 30-day and 1-year mortality risk according to infarct size, stratified by study period.

**Figure 3:**
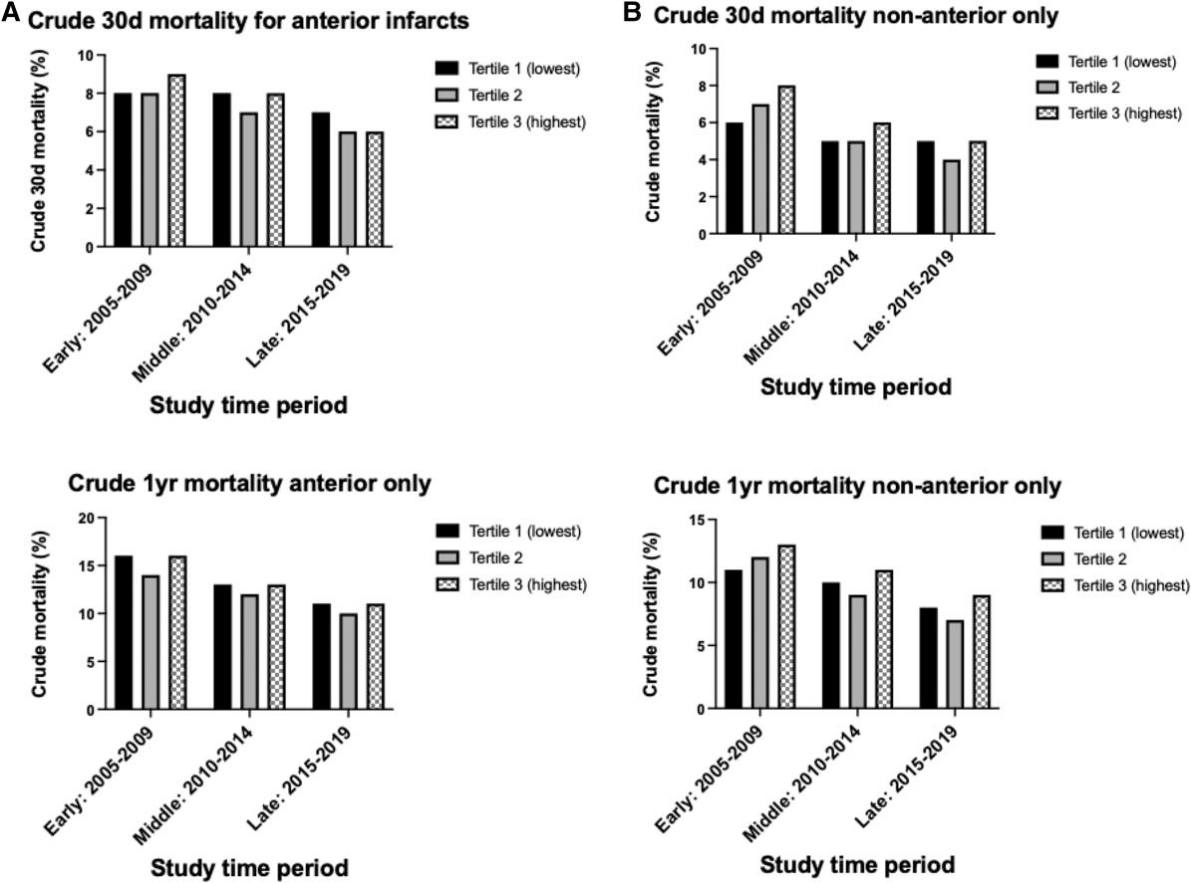
Unadjusted 30-day and 1-year mortality according to infarct site and infarct size. (*A*) Anterior infarction only. (*B*) Non-anterior infarction only.

Unadjusted 30-day mortality was higher in the early study period according to infarct size (T1: 7% vs. T3: 9%), whereas it had declined in the middle (T1: 6% vs. T3: 7%) and late periods (T1: 6% vs. T3: 6%). Unadjusted 1-year mortality declined over the study period: early (T1: 13% vs. T3: 14%), middle (T1: 11% vs. T3: 12%), and late (T1: 9% vs. T3: 10%) (*Table 2* and *Figure 2*). Unadjusted mortality was significantly higher for anterior and non-anterior infarcts in the early study period (2005–09), but both 30-day and 1-year mortality have declined over the study period for both infarct types (*Figure 3*).

### Adjusted clinical outcomes according to infarct size and time period

**Figure 4:**
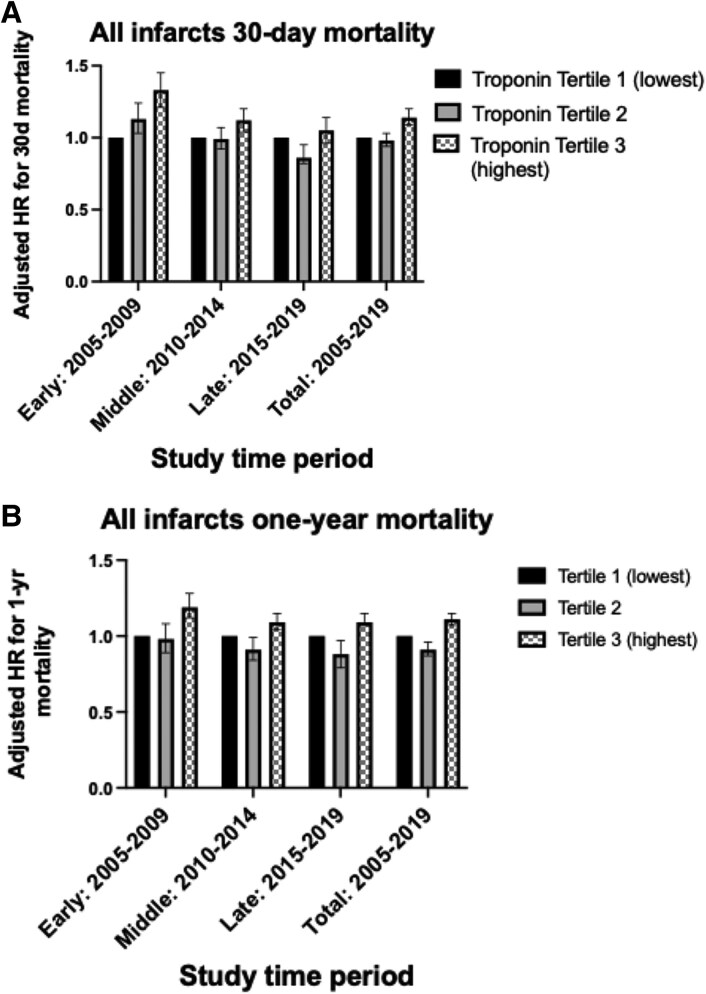
Forest plot of adjusted 30-day and 1-year mortality risk according to infarct size, stratified by study period. (*A*) Thirty-day mortality. (*B*) One-year mortality. Adjusted hazard ratios are presented with 95% CIs. Hazard ratios adjusted for age at admission, gender, year of study, admission heart rate, admission systolic blood pressure, admission hospital region, comorbidities to include prior diagnosis of peripheral vascular disease, previous stroke, previous acute myocardial infarction, previous PCI, previous CABG, prior diagnosis (angina, diabetes mellitus, CKD), family history of coronary artery disease, smoking and asthma or COPD, whether taking warfarin, and whether experienced cardiac arrest either out of hospital or inhospital. Killip classification and ethnicity were not included in models as data were not routinely collected pre-2010.

**Figure 5:**
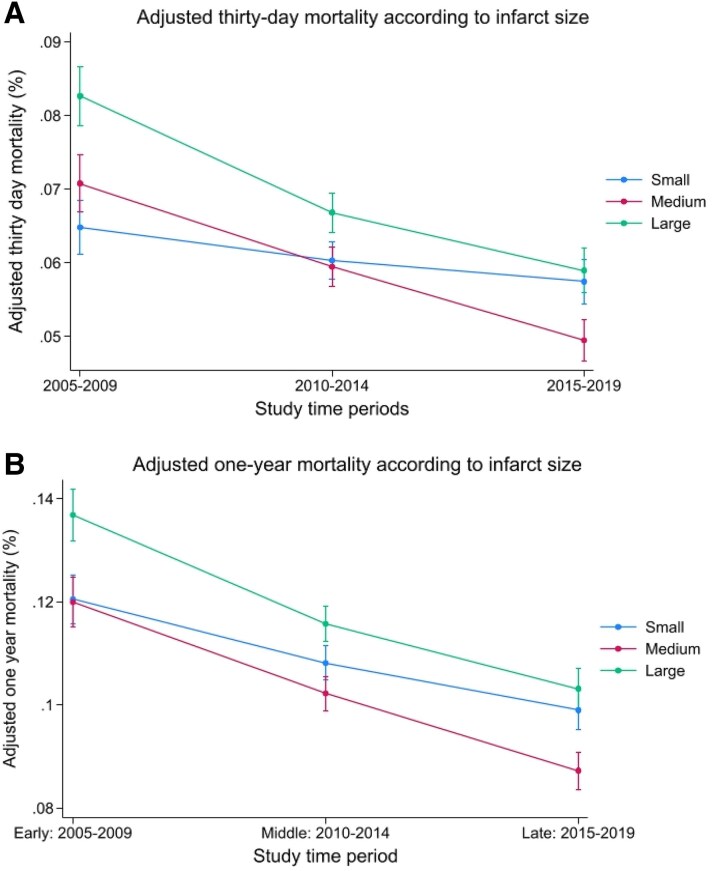
Adjusted 30-day and 1-year mortality temporal trends from the post-estimation model. (*A*) Adjusted 30-day mortality according to infarct size. (*B*) Adjusted 1-year mortality according to infarct size. Thirty-day and 1-year mortality adjusted for age at admission, gender, year of study, admission heart rate, admission systolic blood pressure, admission hospital region, comorbidities to include prior diagnosis of peripheral vascular disease, previous stroke, previous acute myocardial infarction, previous PCI, previous CABG, prior diagnosis (angina, diabetes mellitus, CKD), family history of coronary artery disease, smoking and asthma or COPD, whether taking warfarin, and whether experienced cardiac arrest either out of hospital or in-hospital.

Adjusted risk of 30-day mortality for patients with all infarct types was highest when comparing smallest to largest infarcts in the early study period (adjusted hazard ratio, aHR) (aHR: 1.33, 1.21–1.45, *P* < 0.001), compared to the middle (aHR: 1.12, 1.04–1.15, *P* = 0.002) and not significant in the late study period (aHR: 1.05, 0.96–1.14, *P* = 0.299) (*Table 3* and *Figure 4*). The adjusted risk of mortality at 1 year was higher in larger infarcts in early (aHR: 1.19, 1.12–1.28, *P* < 0.001), middle (aHR: 1.09, 1.04–1.15, *P* = 0.001), and late periods (aHR: 1.07, 1.00–1.15, *P* = 0.046). In the early study period, there was a clear relationship between increasing infarct size and adjusted 30-day mortality, with no overlap of confidence intervals; however, in the middle and late periods, there was significant overlap of groups and confidence intervals (*Figure 5A*). A similar relationship is noted in *Figure 5B* for adjusted 1-year mortality for the largest infarct group in the early study period, but there was no significant separation of Tertiles 1 and 2.

**Table 3:** Adjusted hazard ratios for 30-day and 1-year mortality according to infarct size across study periods

Variables	Admission with STEMI 2005–09 (early)				Admission with STEMI 2010–14 (middle)				Admission with STEMI 2015–19 (late)				Total study period (2005–19)			
	Thirty-day mortality (95% CIs)	*P*-value	One-year mortality (95% CIs)	*P*-value	Thirty-day mortality (95% CIs)	*P*-value	One-year mortality (95% CIs)	*P*-value	Thirty-day mortality (95% CIs)	*P*-value	One-year mortality (95% CIs)	*P*-value	Thirty-day mortality (95% CIs)	*P*-value	One-year mortality (95% CIs)	*P*-value
Tertile 1 (smallest peak troponin)	1.00	N/A	1.00	N/A	1.00	N/A	1.00	N/A	1.00	N/A	1.00	N/A	1.00	N/A	1.00	N/A
Tertile 2	1.13 (1.03–1.23)	0.012	1.01 (0.94–1.08)	0.866	0.99 (0.92–1.07)	0.830	0.95 (0.90–1.00)	0.044	0.86 (0.78–0.94)	0.001	0.87 (0.81–0.93)	<0.001	0.98 (0.93–1.02)	0.320	0.93 (0.90–0.97)	<0.001
Tertile 3 (highest peak)	1.32 (1.21–1.45)	<0.001	1.19 (1.11–1.27)	<0.001	1.12 (1.04–1.20)	0.002	1.09 (1.04–1.15)	0.001	1.05 (0.96–1.15)	0.252	1.07 (1.00–1.15)	0.037	1.14 (1.09–1.20)	<0.001	1.10 (1.07–1.14)	<0.001

Tertile 1 (smallest peak troponin) is used as a reference for multivariate Cox regression models. Hazard ratios adjusted for age at admission, gender, year of study, admission heart rate, admission systolic blood pressure, admission hospital region, comorbidities to include prior diagnosis of peripheral vascular disease, previous stroke, previous acute myocardial infarction, previous PCI, previous CABG, prior diagnosis of angina, diabetes mellitus, CKD, family history of coronary artery disease, smoking and asthma or COPD, whether taking warfarin, and whether experienced cardiac arrest either out of hospital or in-hospital. Killip classification and ethnicity not included in models as data is not routinely collected pre-2010. Early study period refers to 2005–09, middle study period to 2010–14, and late study period to 2015–19.

### Subgroup analysis according to infarct location and size over the course of the study

**Figure 6:**
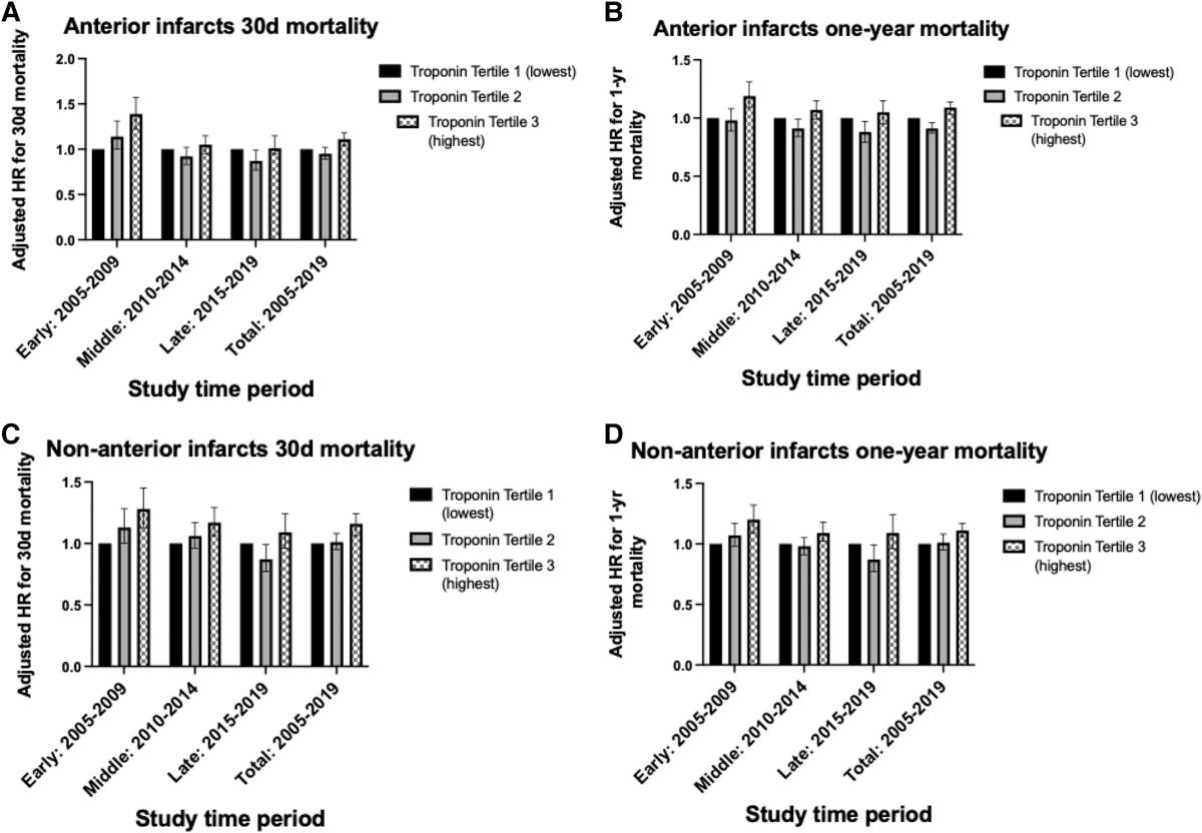
Forest plot of adjusted 30-day and 1-year mortality risk according to infarct size and location, according to study period. (*A*) Anterior infarcts only 30-day mortality. (*B*) Anterior infarcts only 1-year mortality. (*C*) Non-anterior infarcts only 30-day mortality. (*D*) Non-anterior infarcts only 1-year mortality.

The relationship between infarct size and risk of 30-day (aHR: 1.39, 1.22–1.57, *P* < 0.001) and 1-year mortality (aHR: 1.19, 1.09–1.31, *P* < 0.001) was significant for patients with anterior infarction in the early study period, but not significant in the middle or later study periods (*Figure 6* and Supplementary material online, *Table S1*). For non-anterior infarction, infarct size was associated with a higher risk of mortality across for 30-day mortality for early (aHR: 1.28, 1.13–1.45, *P* < 0.001) and middle (aHR: 1.17, 1.06–1.29, *P* = 0.002) but not late (aHR: 1.09, 0.96–1.24, *P* = 0.180) (*Figure 6* and Supplementary material online, *Table S2*).

### Subgroup analysis according to peak troponin assay as a continuous variable

Troponin assay practice changed significantly over the study period; in the early period, this was predominantly troponin I (58–60%) or troponin T (40–42%), while in the middle period, troponin I (46–49%), troponin T (18–19%), hs-TnT (3%), and hs-TnI (30–32%) and troponin I (20–21%), troponin T (7%), hs-TnT (22%), and hs-TnI (50–51%); all assays were used to varying extents. An incremental 10-unit increase in troponin T was associated with an increased risk of 30-day mortality in the early study period (aHR: 1.016, 1.008–1.023, *P* < 0.001), but this was not significant in the middle and later study periods (*Table 4*). There was no significant relationship between an incremental 100-unit increase in hs-TnI across the years of use. A 100-unit increase in hs-TnT was significantly associated with increased 30-day mortality across the total study period (aHR: 1.001, 1.001–1.002, *P* < 0.001), middle period (aHR: 1.000, 1.000–1.001, *P* = 0.040) of the study, and in the later period (aHR: 1.003, 1.002–1.003, *P* < 0.001).

**Table 4:** Relationship between peak troponin value as a continuous variable and mortality outcomes (2005–19)

Variables	Admission with STEMI 2005–09 (early)				Admission with STEMI 2010–14 (middle)				Admission with STEMI 2015–19 (late)				Total study period (2005–19)			
	Thirty-day mortality (95% CIs)	*P*-value	One-year mortality (95% CIs)	*P*-value	Thirty-day mortality (95% CIs)	*P*-value	One-year mortality (95% CIs)	*P*-value	Thirty-day mortality (95% CIs)	*P*-value	One-year mortality (95% CIs)	*P*-value	Thirty-day mortality (95% CIs)	*P*-value	One-year mortality (95% CIs)	*P*-value
Troponin I	1.016 (1.008–1.023)	<0.001	1.010 (1.003–1.017)	0.002	0.996 (0.991–1.000)	0.052	0.998 (0.995–1.001)	0.281	0.994 (0.980–1.010)	0.525	0.996 (0.985–1.010)	0.553	1.010 (1.002–1.011)	0.009	1.003 (0.999–1.010)	0.094
Troponin T	1.049 (1.014–1.084)	0.004	1.037 (1.010–1.064)	0.006	0.991 (0.980–1.001)	0.092	0.995 (0.987–1.002)	0.198	1.018 (0.933–1.042)	0.158	1.011 (0.992–1.030)	0.244	1.001 (0.993–1.001)	0.746	1.000 (0.995–1.001)	0.783
High-sensitivity Troponin T	N/A	N/A	N/A	N/A	1.000 (1.000–1.001)	0.040	1.000 (1.000–1.001)	0.005	1.003 (1.002–1.003)	<0.001	1.003 (1.002–1.003)	<0.001	1.001 (1.001–1.002)	<0.001	1.001 (1.001–1.002)	<0.001
High-sensitivity Troponin I	N/A	N/A	N/A	N/A	1.000 (0.999–1.000)	0.903	0.999 (0.999–1.000)	0.401	1.000 (0.999–1.000)	0.945	1.000 (0.999–1.000)	0.764	1.000 (0.999–1.000)	0.965	0.999 (0.999–1.000)	0.911

Tertile 1 (smallest peak troponin) is used as a reference for multivariate Cox regression models. Hazard ratios adjusted for age at admission, gender, year of study, admission heart rate, admission systolic blood pressure, admission hospital region, comorbidities to include prior diagnosis of peripheral vascular disease, previous stroke, previous acute myocardial infarction, previous PCI, previous CABG, prior diagnosis of angina, diabetes mellitus, CKD, family history of coronary artery disease, smoking and asthma or COPD, whether taking warfarin, and whether experienced cardiac arrest either out of hospital or in-hospital. Killip classification and ethnicity not included in models as data is not routinely collected pre-2010. Early study period refers to 2005–09, middle study period to 2010–14, and late study period to 2015–19. Adjusted hazard ratios refer to a 10-unit increase in troponin I or troponin T and a 100-unit increase in hs-TnT and hs-TnI.

## Discussion

Our study of over 170 000 patients with STEMI from the UK between 2005 and 2019 offers important insights into the relationship of infarct size and mortality over time. Firstly, we demonstrate that larger infarct size according to peak troponin tertile predicts an increased risk of 30-day mortality after STEMI throughout the early and middle years study period. However, this relationship has weakened over the course of our study, no longer significant in the later years. Importantly, the changing temporal trends of the relationship between infarct size and mortality depend upon infarct location. Anterior infarctions in the early study period showed a strong relationship between infarct severity and mortality, but this was no longer significant at the middle and late periods of the study, whereas for non-anterior infarction, there remained a significant association between infarct size and mortality at 30-days, and for 1-year mortality in the early and middle period of the study, diminishing in the late period.

Overall, our study suggests that the relationship between infarct size and 30-day and 1-year mortality post-STEMI has changed over the study period, with this being a strong association of larger infarction and premature mortality in the early study period, but with this relationship weakening over the study years.

Previous studies in this area have important limitations, highlighting the importance of our study. Prior studies using biomarkers such as troponin I and high-sensitivity troponin assays,^5,6^ alongside imaging modalities such as CMRI to assess infarct size, have typically been significantly limited in patient number.^7,11^ Our population-based study is an important step in the understanding of the relationship between infarct size and mortality from STEMI and how this has changed over time.

Thirty-day and 1-year mortality from STEMI has progressively declined since the late 1990s, with increasing rates of GDMT prescribed post-STEMI, such as ACE inhibitors and beta-blockers, and widespread implementation of PPCI networks replacing thrombolysis.^2,3^ This is shown in our study, with progressive improvement in survival between 2005 and 2019, seen in parallel with increases in PPCI rates and GDMT prescription. Additionally, we must consider changes in antiplatelet practice over the study, with increasing usage of prasugrel and ticagrelor in place of clopidogrel, well demonstrated to reduce rates of MACE and stent thrombosis.^12,13^ Cardiac biomarkers as a surrogate marker of infarct size have been reported previously in cohort studies (specifically hs-TnT), where higher peak troponin values were associated with significantly higher 3-year mortality.^5,6^ Where our study adds to the literature is that this relationship is stronger in the historical study period and weaker in the more contemporary era. This observation is supported by Bagai *et al*.^14^ in 2016, who demonstrated in an NSTEMI cohort a significant relationship between peak troponin and long-term mortality in patients that did not undergo revascularization, but no significant differences in mortality according to peak troponin in the revascularized cohort. Similarly, Cediel *et al*.^15^ and Loutati *et al*.^16^ have shown that the newer generation high-sensitivity troponin assays are not correlated with MACE or 1-year post-STEMI mortality in recent years. This is not unexpected, given our understanding of the linear association of time to reperfusion and infarct size,^17^ and we suggest that the advances in PPCI in our study period are a contributor to the reduced importance of infarct size on mortality. This is in keeping with the results of 10 pooled RCTs of PCI for STEMI from the USA, where infarct size had no significant impact on 1-year mortality from STEMI,^18^ although it must be noted that the studies included ranged from 2002 to 2011. Interestingly, Hara *et al*.^19^ demonstrated in the Japanese population that peak high-sensitivity troponin was correlated to poorer long-term survival at 5 years and acknowledged that our curtailing of follow-up to 1-year maximum could be missing important prognostic impacts further from the index infarct. Additionally, by using tertiles, we may be missing a relationship between significant infarctions with increases of up to >10 000 times the upper limit of normal (ULN). McIlvennan *et al*.^20^ showed recently how the risk of 1-year mortality was significantly higher in the >10 000 times ULN troponin rise in AMI when compared to rises below this magnitude, which could explain why we still demonstrate an association between incremental increase in hs-TnT and 30-day mortality throughout the latter years of the study.

An important consideration in this study is the use of troponin assays as surrogate markers for infarct size, which was a pragmatic decision for our study given the absence of CMRI data. While biomarker usage was practical for our analysis, it is important to recognize that in recent years, cardiac magnetic resonance imaging (CMR) has emerged as the gold standard for quantifying infarct size. CMR-derived infarct size has been strongly linked to both all-cause mortality and heart failure readmissions, as demonstrated in a 2016 meta-analysis of post-primary PCI patients.^7^ One advantage of CMRI-based studies of infarct size is that they often evaluate the size of infarct later post-STEMI, Lonborg *et al*.^21^, for example, assessed final infarct size by CMRI at three months, showing a strong association between larger infarcts and increased risk of all-cause mortality and heart failure hospitalization.

Reassuringly for our study, there is good evidence that peak cardiac troponin level correlates well with both CMRI infarct size and LVEF,^22,23^ although there is some suggestion that slightly later troponin measurements, such as up to 4 days post-infarct, may correlate better with CMRI findings.^24^ One challenge from our study is that we do not have the timing of the peak troponin, but based on typical clinical practice in the UK, these are most often both taken in the first 24 h of admission, rather than later in the admission. An additional concern with biomarkers is that peak troponin is not solely dependent on the severity of acute infarction, with comorbidities such as CKD and intercurrent illnesses being associated with a higher peak troponin level,^25^ whereas CMRI infarct size assessment will better reflect infarction size without significant impact from multimorbidity. The lack of correlation with CMRI could mean that cohorts formed from peak troponin level and from CMRI infarct size are not entirely the same populations, given the different timings of CMRI assessment and biomarker measurements, and that further research could be undertaken to see whether our study findings are replicated in STEMI patients with infarct size categorized by CMRI.

An important part of our results is the consideration of the relationship between infarct severity and mortality according to infarct location, an area that has not been as well covered in the literature. Anterior infarctions are associated with the highest in-hospital and long-term mortality, complicated by higher rates of cardiogenic shock and poorer LV function.^4,26^ Our study has shown that over time, the relationship between infarct size and mortality has diminished for anterior infarcts but more slowly for non-anterior infarcts. We suspect that anterior infarctions, known to result in greater myonecrosis,^27^ with higher mortality rates, obtain a greater benefit from primary PCI than smaller, non-anterior infarcts. With primary PCI rates significantly increasing over the study, this could be a contributor to our results. It is interesting to consider whether, with the heterogeneity of non-anterior infarction, cardiac biomarkers do still have an important role in risk stratification of infarcts. Nevertheless, it is still important to acknowledge that overall, in keeping with recent US data, anterior infarcts remain the highest mortality infarcts in STEMI. One marked change in practice over the study periods is the increased rates of prescription of aldosterone antagonists, especially in patients with large infarct sizes. With convincing evidence for improved mortality post-STEMI with aldosterone antagonists, this could be a mechanism for the improvements in mortality in patients with anterior infarction, alongside reducing the difference in survival according to infarct size.^28^ Although not captured by MINAP, we suggest that advances in contemporary heart failure therapy are additionally improving the survival of those with the largest infarcts. Over our study period, there will have been significant increases in the proportion of patients receiving angiotensin receptor–neprilysin inhibitors (ARNIs), for example, which have been well established to reduce HF hospitalizations and cardiac death in patients with reduced LV function.^29,30^ Sodium–glucose cotransporter-2 inhibitors (SGLT2) have proliferated in use in patients with impaired LV function in recent years, with reductions in cardiovascular death and heart failure hospitalizations demonstrated across the spectrum of LV function,^31,32^ and suggestion of benefit in patients post-PCI for AMI.^33^ Patients with the largest infarctions will be more likely to be receive earlier titration of heart failure therapy with ARNIs and SGLT2 inhibitors, which we suggest will be a cause of the declining relationship between infarct size and early STEMI mortality. Furthermore, we must also consider the impact of the changes in cardiac device therapy over the study period, and whether this could be reducing the relationship between infarct size and mortality. Firstly, there has been increasing focus on primary prevention implantable cardioverter defibrillator (ICD) where LV function remains impaired post-STEMI,^34^ although we suggest this will not be relevant for 30-day mortality, as these devices will typically be implanted beyond six weeks, and these could be influencing 1-year mortality. Additionally, there is increasing implantation of cardiac resynchronization therapy defibrillators or pacemakers in those with impaired ventricular function, which we suggest could further reduce the link between infarct size and 1-year mortality.^35^

Finally, we must also consider the role of systemic improvements in provision of speciality cardiology care that will have taken place over the study period, with increased awareness of managing STEMI in coronary care or intensive care units, for example, which we expect the larger infarcts will be prioritized for.^36^ Overall, we suggest that the reduced association of infarct size and mortality seen in our study is a complex, multifactorial process, with contributions from increased availability and reduced reperfusion time of PPCI resulting from regional PPCI networks, improvements in contemporary PCI techniques, and improvements in GDMT, including both antiplatelet agents and heart failure pharmacotherapy and cardiac device therapy such as primary prevention ICD and CRTs for those with impaired LV function.

### Strengths

This study has several strengths. Firstly, the MINAP registry captures a comprehensive range of data from every STEMI admission across the UK. Such a large dataset, from a universal healthcare system, can give important insights into the temporal trends and contemporary care of STEMI patients, more generalizable to the wider population. Additionally, our comprehensive mortality linkage to the ONS registration of deaths means that we capture all aspects of all-cause mortality, be that in the index hospital, community, or an alternate hospital, enabling accurate analysis of mortality outcomes at our specified time periods.

### Limitations

This study includes several limitations. Firstly, despite the broad number of variables included in MINAP, the nature of observational studies of this kind is that additional important confounders that could have mediated outcomes, such as frailty and coronary artery disease severity, are not captured and cannot be included in our models. Therefore, there is a risk of residual confounding affecting the interpretation of our results.

Specifically, there was a significant proportion of patients with missing data for Killip classification, which was not routinely collected in the early study period, therefore not included in our adjusted models. This could be important in the interpretation of our results, as patients admitted in the early study may have been presenting in more advanced stages of heart failure. Furthermore, there remains a significant proportion of missing data in LV function assessment post-STEMI, alongside the absence of any additional detailed metrics of heart failure, alongside a lack of data regarding titration of heart failure therapy, especially newer agents such as SGLT2 inhibitors or ARNIs. More comprehensive data regarding the degree of LV dysfunction, the extent of heart failure severity, and heart failure pharmacotherapy would have significantly aided our manuscript and our understanding as to whether contemporary heart failure therapy is a key driver of our results.

MINAP should capture data on Type 1 AMI only; however, data is subject to misclassification; therefore, a small number of patients with Type 2 MI may be included. Missing data is another inherent issue in large-scale registry studies; we did not include patients with missing peak troponin level, troponin assay, and infarct site data, which could affect the generalizability of our results. Additionally, we acknowledge that most frequently used troponin assays have changed over the study period, with a change to higher sensitivity assays towards the latter end of our study, and separate assays may have differing ability to estimate infarct size, and our results must be interpreted in the context of this. Troponin values can be higher in revascularized patients and exhibit a second peak following successful reperfusion, with higher sensitivity troponin assays and higher rates of reperfusion towards the end of the study; this could contribute to the weakening association between peak troponin and mortality.^14,37^ We acknowledge that troponin values should be interpreted in the context of renal function, and although we have included CKD as a covariate in our survival models, due to a significant proportion of missing data in serum creatinine, we have not been able to adjust individual troponin values according to serum creatinine levels. Finally, MINAP does not capture data regarding whether patients have left ventricular hypertrophy (LVH), which has been shown to predict greater troponin release, which can lead to overestimation of infarct size.^38^ Finally, it is important to interpret our results in the context of us not being able to determine the timing of the peak troponin value. There could be patients included in the smallest peak troponin tertile where this value was taken early in the trajectory of STEMI, for example, and not repeated after PPCI, where it may have been substantially higher later. There could therefore be high-risk patients included in Tertile 1, which could give a reason for why we see slightly lower adjusted mortality in Tertile 2 compared to 1 in the late study period only.

## Conclusions

In a large, national STEMI registry, we show the improvements in 30-day and 1-year mortality from STEMI between 2005 and 2019. Infarct size according to peak troponin level tertiles predicted 30-day mortality over the early (2005–09) and middle (2010–14) study period; this relationship was far stronger in the early (2005–09) years of our study but was no longer significant in the late study period (2015–19). The relationship between infarct size and mortality was no longer present between 2010 and 2019 for anterior infarction, whereas this remained for non-anterior infarcts up to 2015, suggesting the non-anterior infarcts are a future area for optimization of STEMI care.

## Supplementary Material

oeaf111_Supplementary_Data

## Data Availability

The authors do not have authorization to share the data, but it can be accessed through contacting the National Institute for Cardiovascular Outcomes Research (NICOR) upon approval.
