# Supplementary material for: The temporal trends of ST-elevation myocardial infarction mortality according to infarct size and location: insights from the UK National MINAP registry from 2005 to 2019
Source: Eur Heart J Open. 2025 Aug 20;5(5):oeaf111. doi: 10.1093/ehjopen/oeaf111 (PMC12448480; doi:10.1093/ehjopen/oeaf111)
Supplement: oeaf111_Supplementary_Data [file oeaf111_supplementary_data.docx]

**Supplementary resources**

**Supplementary Table 1: Adjusted mortality outcomes for all study patients with anterior infarct only from 2005-2019**

| **Variables** | **Admission with STEMI 2005-2009 (early)** | | | | **Admission with STEMI 2010-2014 (middle)** | | | | **Admission with STEMI 2015-2019 (late)** | | | | **Total Study Period (2005-2019)** | | | |
| --- | --- | --- | --- | --- | --- | --- | --- | --- | --- | --- | --- | --- | --- | --- | --- | --- |
|  | **Thirty-day mortality (95% CIs)** | **P-value** | **One-year mortality (95% CIs)** | **P-value** | **Thirty-day mortality (95% CIs)** | **P-value** | **One-year mortality (95% CIs)** | **P-value** | **Thirty-day mortality (95% CIs)** | **P-value** | **One-year mortality (95% CIs)** | **P-value** | **Thirty-day mortality (95% CIs)** | **P-value** | **One-year mortality (95% CIs)** | **P-value** |
| **Tertile 1 (smallest peak troponin)** | 1.00 | N/A | 1.00 | N/A | 1.00 | N/A | 1.00 | N/A | 1.00 | N/A | 1.00 | N/A | 1.00 | N/A | 1.00 | N/A |
| **Tertile 2** | 1.14 (1.00-1.31) | 0.048 | 0.98 (0.89-1.08) | 0.675 | 0.92 (0.83-1.02) | 0.117 | 0.91 (0.84-0.99) | 0.021 | 0.87 (0.77-0.99) | 0.042 | 0.88 (0.79-0.97) | 0.012 | 0.95 (0.89-1.02) | 0.177 | 0.91 (0.87-0.96) | 0.001 |
| **Tertile 3 (Highest peak)** | 1.39 (1.22-1.57) | <0.001 | 1.19 (1.09-1.31) | <0.001 | 1.05 (0.95-1.15) | 0.381 | 1.07 (0.99-1.15) | 0.092 | 1.01 (0.89-1.15) | 0.862 | 1.05 (0.95-1.15) | 0.358 | 1.11 (1.04-1.18) | 0.002 | 1.09 (1.03-1.14) | 0.001 |

**Supplementary Table 2: Adjusted mortality outcomes for all study patients with non-anterior infarct only from 2005-2019**

| **Variables** | **Admission with STEMI 2005-2009 (early)** | | | | **Admission with STEMI 2010-2014 (middle)** | | | | **Admission with STEMI 2015-2019 (late)** | | | | **Total Study Period (2005-2019)** | | | |
| --- | --- | --- | --- | --- | --- | --- | --- | --- | --- | --- | --- | --- | --- | --- | --- | --- |
|  | **Thirty-day mortality (95% CIs)** | **P-value** | **One-year mortality (95% CIs)** | **P-value** | **Thirty-day mortality (95% CIs)** | **P-value** | **One-year mortality (95% CIs)** | **P-value** | **Thirty-day mortality (95% CIs)** | **P-value** | **One-year mortality (95% CIs)** | **P-value** | **Thirty-day mortality (95% CIs)** | **P-value** | **One-year mortality (95% CIs)** | **P-value** |
| **Tertile 1 (smallest peak troponin)** | 1.00 | N/A | 1.00 | N/A | 1.00 | N/A | 1.00 | N/A | 1.00 | N/A | 1.00 | N/A | 1.00 | N/A | 1.00 | N/A |
| **Tertile 2** | 1.13 (1.00-1.28) | 0.048 | 1.07 (0.98-1.17) | 0.153 | 1.06 (0.96-1.17) | 0.264 | 0.98 (0.91-1.05) | 0.514 | 0.87 (0.77-0.99) | 0.032 | 0.87 (0.77-0.99) | 0.032 | 1.01 (0.95-1.08) | 0.788 | 0.97 (0.92-1.02) | 0.178 |
| **Tertile 3 (Highest peak)** | 1.28 (1.13-1.45) | <0.001 | 1.20 (1.09-1.32) | <0.001 | 1.17 (1.06-1.29) | 0.002 | 1.09 (1.02-1.18) | 0.016 | 1.09 (0.96-1.24) | 0.180 | 1.09 (0.96-1.24)-1.22) | 0.180 | 1.16 (1.09-1.24) | <0.001 | 1.11 (1.06-1.17) | <0.001 |

**Supplementary Table 3:** **Adjusted hazard ratios for thirty-day and one-year mortality according to infarct size across study period for patients that underwent reperfusion and with complete data for total reperfusion time, adjusted for whether underwent PPCI or thrombolysis, and whether total ischaemic time was under 120 minutes**

| **Variables** | **Admission with STEMI 2005-2009 (early)** | | | | **Admission with STEMI 2010-2014 (middle)** | | | | **Admission with STEMI 2015-2019 (late)** | | | | **Total Study Period (2005-2019)** | | | |
| --- | --- | --- | --- | --- | --- | --- | --- | --- | --- | --- | --- | --- | --- | --- | --- | --- |
|  | **Thirty-day mortality (95% CIs)** | **P-value** | **One-year mortality (95% CIs)** | **P-value** | **Thirty-day mortality (95% CIs)** | **P-value** | **One-year mortality (95% CIs)** | **P-value** | **Thirty-day mortality (95% CIs)** | **P-value** | **One-year mortality (95% CIs)** | **P-value** | **Thirty-day mortality (95% CIs)** | **P-value** | **One-year mortality (95% CIs)** | **P-value** |
| **Tertile 1 (smallest peak troponin)** | 1.00 | N/A | 1.00 | N/A | 1.00 | N/A | 1.00 | N/A | 1.00 | N/A | 1.00 | N/A | 1.00 | N/A | 1.00 | N/A |
| **Tertile 2** | 0.86 (0.63-1.16) | 0.320 | 0.92 (0.75-1.12) | 0.410 | 0.92 (0.81-1.04) | 0.182 | 0.90 (0.82-0.98) | 0.018 | 0.80 (0.72-0.90) | <0.001 | 0.84 (0.77-0.91) | <0.001 | 0.84 (0.78-0.92) | <0.001 | 0.86 (0.81-0.92) | <0.001 |
| **Tertile 3 (Highest peak)** | 1.40 (1.08-1.82) | 0.012 | 1.38 (1.15-1.64) | <0.001 | 1.24 (1.11-1.40) | <0.001 | 1.20 (1.10-1.30) | <0.001 | 1.07 (0.96-1.19) | 0.235 | 1.13 (1.04-1.22) | 0.004 | 1.15 (1.07-1.24) | <0.001 | 1.17 (1.11-1.24) | <0.001 |

Tertile 1 (smallest peak troponin) is used as reference for multivariate cox-regression models. Hazard ratios adjusted for: Age at admission, gender, admission heart rate, admission systolic blood pressure, admission hospital region, comorbidities to include prior diagnosis of peripheral vascular disease, previous stroke, previous acute myocardial infarction, previous PCI, previous CABG, prior diagnosis of angina, diabetes mellitus, CKD, family history of coronary artery disease, smoking and asthma or COPD, whether taking warfarin, whether underwent primary percutaneous coronary intervention or thrombolysis, whether total ischaemic time was under 120 minutes, and whether experienced cardiac arrest either out of hospital or in-hospital. Killip classification and ethnicity not included in models as data not routinely collected pre-2010. Early study period refers to 2005-2009, middle study period is 2010-2014 and late study period is 2015-2019.

**Supplementary Table 4:** **Adjusted hazard ratios for thirty-day and one-year mortality according to infarct size across study period for patients that underwent reperfusion and with complete data for total reperfusion time for men only**

| **Variables** | **Admission with STEMI 2005-2009 (early)** | | | | **Admission with STEMI 2010-2014 (middle)** | | | | **Admission with STEMI 2015-2019 (late)** | | | | **Total Study Period (2005-2019)** | | | |
| --- | --- | --- | --- | --- | --- | --- | --- | --- | --- | --- | --- | --- | --- | --- | --- | --- |
|  | **Thirty-day mortality (95% CIs)** | **P-value** | **One-year mortality (95% CIs)** | **P-value** | **Thirty-day mortality (95% CIs)** | **P-value** | **One-year mortality (95% CIs)** | **P-value** | **Thirty-day mortality (95% CIs)** | **P-value** | **One-year mortality (95% CIs)** | **P-value** | **Thirty-day mortality (95% CIs)** | **P-value** | **One-year mortality (95% CIs)** | **P-value** |
| **Tertile 1 (smallest peak troponin)** | 1.00 | N/A | 1.00 | N/A | 1.00 | N/A | 1.00 | N/A | 1.00 | N/A | 1.00 | N/A | 1.00 | N/A | 1.00 | N/A |
| **Tertile 2** | 1.13 (1.00-1.28) | 0.052 | 1.00 (0.92-1.10) | 0.875 | 0.97 (0.88-1.07) | 0.569 | 0.94 (0.87-1.01) | 0.097 | 0.83 (0.74-0.93) | 0.002 | 0.86 (0.79-0.94) | 0.001 | 0.96 (0.90-1.02) | 0.169 | 0.93 (0.88-0.97) | 0.002 |
| **Tertile 3 (Highest peak)** | 1.26 (1.12-1.42) | <0.001 | 1.12 (1.02-1.23) | 0.013 | 1.05 (0.96-1.15) | 0.295 | 1.07 (1.00-1.15) | 0.047 | 0.97 (0.87-1.09) | 0.614 | 1.01 (0.93-1.10) | 0.749 | 1.07 (1.00-1.13) | 0.043 | 1.06 (1.01-1.11) | 0.019 |

**Tertile 1 (smallest peak troponin) is used as reference for multivariate cox-regression models. Hazard ratios adjusted for: Age at admission, admission heart rate, admission systolic blood pressure, admission hospital region, comorbidities to include prior diagnosis of peripheral vascular disease, previous stroke, previous acute myocardial infarction, previous PCI, previous CABG, prior diagnosis of angina, diabetes mellitus, CKD, family history of coronary artery disease, smoking and asthma or COPD, whether taking warfarin, and whether experienced cardiac arrest either out of hospital or in-hospital. Killip classification and ethnicity not included in models as data not routinely collected pre-2010.**

**Supplementary Table 5:** **Adjusted hazard ratios for thirty-day and one-year mortality according to infarct size across study period for patients that underwent reperfusion and with complete data for total reperfusion time for women only**

| **Variables** | **Admission with STEMI 2005-2009 (early)** | | | | **Admission with STEMI 2010-2014 (middle)** | | | | **Admission with STEMI 2015-2019 (late)** | | | | **Total Study Period (2005-2019)** | | | |
| --- | --- | --- | --- | --- | --- | --- | --- | --- | --- | --- | --- | --- | --- | --- | --- | --- |
|  | **Thirty-day mortality (95% CIs)** | **P-value** | **One-year mortality (95% CIs)** | **P-value** | **Thirty-day mortality (95% CIs)** | **P-value** | **One-year mortality (95% CIs)** | **P-value** | **Thirty-day mortality (95% CIs)** | **P-value** | **One-year mortality (95% CIs)** | **P-value** | **Thirty-day mortality (95% CIs)** | **P-value** | **One-year mortality (95% CIs)** | **P-value** |
| **Tertile 1 (smallest peak troponin)** | 1.00 | N/A | 1.00 | N/A | 1.00 | N/A | 1.00 | N/A | 1.00 | N/A | 1.00 | N/A | 1.00 | N/A | 1.00 | N/A |
| **Tertile 2** | 1.11 (0.97-1.26) | 0.140 | 1.00 (0.91-1.11) | 0.980 | 1.01 (0.91-1.13) | 0.797 | 0.95 (0.88-1.03) | 0.228 | 0.90 (0.77-1.05) | 0.166 | 0.89 (0.79-0.99) | 0.040 | 1.00 (0.93-1.08) | 0.969 | 0.94 (0.89-1.00) | 0.038 |
| **Tertile 3 (Highest peak)** | 1.42 (1.25-1.63) | <0.001 | 1.29 (1.17-1.43) | <0.001 | 1.21 (1.09-1.35) | <0.001 | 1.12 (1.03-1.21) | 0.005 | 1.20 (1.04-1.40) | 0.014 | 1.19 (1.06-1.33) | 0.003 | 1.26 (1.17-1.35) | <0.001 | 1.18 (1.12-1.24) | <0.001 |

**Tertile 1 (smallest peak troponin) is used as reference for multivariate cox-regression models. Hazard ratios adjusted for: Age at admission, admission heart rate, admission systolic blood pressure, admission hospital region, comorbidities to include prior diagnosis of peripheral vascular disease, previous stroke, previous acute myocardial infarction, previous PCI, previous CABG, prior diagnosis of angina, diabetes mellitus, CKD, family history of coronary artery disease, smoking and asthma or COPD, whether taking warfarin, and whether experienced cardiac arrest either out of hospital or in-hospital. Killip classification and ethnicity not included in models as data not routinely collected pre-2010.**

**Supplementary Table 6: Adjusted cox-regression model with interaction term for peak troponin tertile and study period**

| **Variables** | **Adjusted Hazard ratio for 30-day mortality with 95% CIs** | **P-value** |
| --- | --- | --- |
| **Overall Tertile:** |  |  |
| Medium | 1.13 (1.03-1.23) | 0.011 |
| Large | 1.33 (1.22-1.46) | 0.001 |
|  |  |  |
| **Year Group:** |  |  |
| Middle (2010-2014) | 0.94 (0.86-1.02) | 0.115 |
| Late (2015-2019) | 0.89 (0.81-0.98) | 0.013 |
|  |  |  |
| ***Interaction term:** |  |  |
| Medium # middle | 0.88 (0.78-0.98) | 0.024 |
| Medium # late | 0.74 (0.65-0.84) | <0.001 |
| Large # middle | 0.83 (0.75-0.93) | 0.001 |
| Large # late | 0.77 (0.68-0.87) | <0.001 |

**Hazard ratios additionally adjusted for: Age at admission, admission heart rate, admission systolic blood pressure, admission hospital region, comorbidities to include prior diagnosis of peripheral vascular disease, previous stroke, previous acute myocardial infarction, previous PCI, previous CABG, prior diagnosis of angina, diabetes mellitus, CKD, family history of coronary artery disease, smoking and asthma or COPD, whether taking warfarin, and whether experienced cardiac arrest either out of hospital or in-hospital. Killip classification and ethnicity not included in models as data not routinely collected pre-2010.**

- Pairwise comparisons for the effect of medium or large troponin tertiles (versus small tertile) between the Year group of 2005-2009 and other study year groups.

**Supplementary Table 7: Sensitivity analysis for patients with peak troponin from hs-TnT assay only between 2015-2019**

| **Variables** | **Admission with STEMI 2015-2019 (late) with peak hs-TNT only** | | | |
| --- | --- | --- | --- | --- |
|  | **Thirty-day mortality (95% CIs)** | **P-value** | **One-year mortality (95% CIs)** | **P-value** |
| **Tertile 1 (smallest peak troponin)** | 1.00 | N/A | 1.00 | N/A |
| **Tertile 2** | 0.72 (0.63-0.83) | <0.001 | 0.80 (0.73-0.89) | <0.001 |
| **Tertile 3 (Highest peak troponin)** | 1.01 (0.89-1.14) | 0.861 | 1.12 (1.02-1.22) | 0.023 |
